# Effective Communication Within and Across Public Health Institutions: A Qualitative Study in Switzerland

**DOI:** 10.3389/ijph.2025.1609055

**Published:** 2026-01-08

**Authors:** Maddalena Fiordelli, Nicola Diviani, Alexander Ort, Sara Rubinelli

**Affiliations:** 1 Institute of Public Health, Università della Svizzera italiana, Lugano, Switzerland; 2 Faculty of Health Sciences and Medicine, University of Lucerne, Lucerne, Switzerland; 3 Person‐Centered Healthcare & Health Communication Group, Swiss Paraplegic Research, Nottwil, Switzerland

**Keywords:** COVID-19, effective communication, institutional communication, internal communication, public health communication

## Abstract

**Objectives:**

This paper explores the communication challenges faced by public health institutions in Switzerland during COVID-19 pandemic and identifies key barriers and facilitators of effective communication within and across public health institutions.

**Methods:**

In 2021, we conducted qualitative online semi-structured interviews to collect opinions and experiences of representatives of Swiss public health institutions. Interview transcripts were analyzed using inductive thematic analysis to identify main themes.

**Results:**

Key facilitators of effective communication included flexibility, dedicated resources, new processes, coordination, and experiential knowledge. Inter-institutional communication benefited from clear processes, active listening, mediation, and strong networks. Barriers mirrored these factors, such as role tensions, unclear responsibilities, and limited resources. Discrepancies across cantons and between government levels, especially between the confederation and cantons, often hindered communication.

**Conclusion:**

To strengthen communication in public health crises, we recommend clear protocols, centralized structures, and bridging cultural and linguistic gaps. Ongoing training and diverse perspectives are key to effective communication within and across public health institutions in Switzerland and beyond.

## Introduction

Public health emergencies like the COVID-19 pandemic require strong coordination within and across public health institutions at regional and national levels [1], enabled by effective communication. In Switzerland, with its four linguistic regions, cultural diversity complicates these efforts [2]. The COVID-19 crisis revealed significant communication challenges that hindered the overall crisis response [3]. The absence of clear guidance on institutional and intergovernmental communication [4], even in established emergency risk communication models [5], was particularly problematic in the unprecedented “infodemic” context [6, 7]. This underscores the critical role of effective communication in ensuring timely information sharing, consistent messaging, and coherent action across governance levels.

Effective communication from institutions to the public is essential during emergencies, as it fosters trust and enables coordinated action. Clear, transparent information aligns public behavior with response strategies, reducing confusion and ensuring communities can respond effectively [8–10]. Swiss authorities, reflecting their cultural approach, typically adopt an analytical, measured style, contrasting with the more emotive or directive styles of neighboring countries [11–13]. This fact-based messaging has generally maintained public trust by meeting expectations for transparency and rational discourse. However, misalignments in communication sometimes caused confusion and discontent, underscoring the challenges of Switzerland’s complex governance system [14]. With decision-making powers distributed across institutions, coordination is required both within the federal level—e.g., between departments on health financing and public health measures—and across levels, through bodies such as the Swiss Conference of the Cantonal Ministers of Public Health (GDK/CDS), which harmonizes efforts on specialized medical care and health policy [14].

The Swiss health system is shaped by federalism, which brings both strengths and challenges [14]. Healthcare delivery is efficient and geographically close to the population, yet fragmentation—highlighted by external evaluations—raises concerns about coordination, transparency, and errors [15]. These tensions became visible during COVID-19: despite existing guidance, varied responses across government levels reflected the limits of decentralization [2, 12]. Roles and responsibilities shifted between phases, with changing management plans underscoring the difficulty of coordinating an effective response [3, 11]. While communication is widely recognized as essential in emergencies, the importance of structured intergovernmental coordination is often underestimated. These dynamics also intersect with political trust. Switzerland enjoys comparatively high and stable trust, linked to symmetrical federalism, cantonal autonomy, and direct democracy [16]. Yet past crises, such as the 2009 H1N1 pandemic, showed trust can erode: confidence in the federal government fell due to concerns over transparency, vaccination pressure, and perceived conflicts of interest. These experiences illustrate that even in high-trust, decentralized systems, crisis response depends not only on institutional design but also on intergovernmental collaboration and credible public communication [17].

This paper is part of a larger study aimed at developing a toolkit for health institutions on effective communication during public health emergencies. Specifically, it focuses on the internal communication challenges faced by Swiss institutions during the pandemic, aiming to identify barriers and facilitators to improve future crisis communication strategies.

## Methods

In this qualitative study, we adopted an Interpretative Phenomenological approach to capture the essence of communication during the pandemic, focusing on individuals’ lived experiences and their meanings [18]. This method was chosen for its suitability in exploring nuanced perceptions and contextual dynamics of communication and coordination in a health emergency, offering depth that quantitative methods cannot provide. Data were collected from key public health actors involved in the emergency response from the outset.

### Recruitment

Between the first (March–May 2020) and the second wave (October 2020 – February 2021) we invited via email all relevant public institutions in Switzerland, both at federal and cantonal level, who were dealing with communication during the pandemic. Institutions were included if they were responsible for communicating with the public at the national or cantonal level. Institutions without a communication mandate or without responsibilities related to pandemic communication were excluded. The invitation presented the study aim and procedures and asked for voluntary participation to an online interview to be scheduled upon the interviewee’s preferences. Two reminders were sent.

### Data Collection

We conducted online semi-structured interviews using a study-specific guide (see Supplementary Material). Given Switzerland’s multilingual context, three interviewers (one male, two females) conducted interviews in participants’ mother tongue to ensure cultural sensitivity. No prior relationship was established. Participants were informed of the researchers’ affiliation, study purpose, and the broader project. Interviewers—all PhD-level researchers in health communication with qualitative expertise—discussed potential biases within the team to support reflexivity. The guide included stable themes: a narrative of the first wave, then the second, and finally reflections on their comparison, with additional questions (e.g., infodemic management) asked as relevant.

Interviews took place between January and July 2021, averaging 40 min. Sessions were video-recorded, audio-extracted, and professionally transcribed in both the original language and English. Data collection continued until theoretical saturation was reached, after which sampling was stopped.

### Data Analysis

After familiarizing with the data, we thematically analyzed all transcripts [19]. MF inductively coded the first half of the interviews, allowing themes to emerge, and discussed preliminary codes with ND and SR to ensure shared understanding. These codes were consolidated into two categories: barriers and facilitators of institutional communication. Once data saturation was reached, the remaining interviews were analyzed deductively using the established framework, with themes refined for coherence. Comparison with ND, who analyzed the corpus for another study [20], informed the final labeling of themes presented here.

## Results

Of the 57 public institutions contacted, 6 declined and 28 did not respond. The final sample included 25 representatives from 23 cantonal or federal bodies. At the federal level, five institutions were represented, including communication and media specialists, COVID-19 task force members, and representatives of the federal ethical commission. Cantonal representatives comprised seven health department heads, six cantonal doctors (chief public health officers), and five communication specialists. Most participants were from the German-speaking region (65%), followed by the Italian- (13%) and French-speaking regions (4%). This diversity captured perspectives reflective of Switzerland’s multilingual, decentralized governance, a critical factor for examining communication and coordination in a federal health system.

**FIGURE 1:**
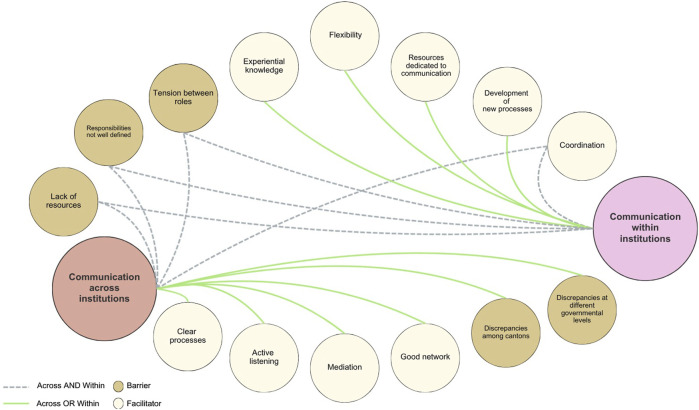
Developing standards for institutional health communication during public health emergencies. Learning from information around COVID-19 pandemic as a case in point (Switzerland, 2020-2023).

Our analysis identified barriers and facilitators in two communication processes [1]: within public health institutions and [2] across institutions (Figure 1). The figure illustrates how these factors interact across the two processes, highlighting where challenges constrain coordination and where supportive conditions strengthen information flow. The arrows indicate whether a factor affects internal communication, cross-institutional collaboration, or both.

### Communication Within the Public Health Institution

All interviewees described the pandemic as hectic, with a high volume and frequency of information exchanged. In this context, federal and cantonal representatives identified five factors that facilitated internal communication: coordination, new processes, dedicated resources, flexibility, and experiential knowledge. They also noted three barriers—role tensions, unclear responsibilities, and lack of resources—which emerged only from cantonal interviews (Figure 1).

#### Facilitators to the Communication Within the Public Health Institution

Many noted that the continuous demands on public institutions can only be managed through coordinated communication, which must occur within and across departments.

*“We also have to coordinate many enquiries, coordinate them between the departments (e.g., Department of Economic Affairs) in one answer. […] And if it weren’t for the central communication office, then there would be wild back-and-forth communication and one contradiction after the other towards the outside.”* (Cantonal representative)

Critical situations require new processes—either pre-established through contingency planning or activated collectively during crises. This view was shared by both cantonal and federal representatives, as illustrated by the following quote from a cantonal participant:

*“Regarding communication? The structure. Courses of action. Crisis procedures. If a huge crisis were to happen, for example, let’s assume that that our government council has secretly had itself vaccinated and the media would find out.”* (Cantonal representative)

Cantonal and federal representatives stressed that dedicated communication resources were crucial for addressing internal challenges. In Switzerland’s multilingual context, the following quote from a cantonal representative of a bilingual canton illustrates the need for tailored resources that reflect cultural and linguistic nuances.

*“Any communication issued by the canton—whether from the health office, the government, or the management staff—passes through this department and is ultimately released by the registry office or the media service. The collaboration now works well. It took considerable effort to reach this point, but it functions effectively today. We have established agreements, and whenever possible, the necessary resources—such as translation services—are reserved in advance*.*”* (Cantonal representative)

Some described flexibility as the best possible response, allowing for resilient management of the challenges. This flexible approach was paired with a courageous attitude and an innovative mindset, which a federal representative emphasized as valuable not only during the crisis but also for future applications.

*“And then it was an ongoing, rolling process. […] And I think the pandemic has shown that when there is pressure to act, you come up with good solutions very quickly, and that you have the courage to implement them because you are forced to do so. And that is something I would generally take away as an important insight, that in today's world - and the greater the challenge, the greater the courage. That courage and the joy of implementation are needed to get things rolling, and that nothing is as classic and static as one thinks, and that one should and can therefore also dare to take new paths. […]”* (Federal Representative)

Finally, when comparing the two waves, many emphasized the importance of experiential knowledge as a key facilitator of communication within public institutions during the pandemic crisis.

*“There is no comparison. Although the second wave was much worse than the first, it went much more calmly and orderly, because they had all these experiences from the first wave and because they knew; now we have to watch out, now we have to react, now we have to pick them up. That went much better.”* (Cantonal representative)

#### Barriers to the Communication Within the Public Health Institution

The first barrier identified was the tension between roles. Different departments and representatives often had conflicting priorities, which made it challenging to maintain effective communication in a stress-free environment. This was highlighted by some cantonal representatives.

*“The health office leads the crisis response in the canton, but it includes different departments that represent different interests. I encounter this tension every day: there is a constant balancing act between economic and public health priorities. The Director of Economic Affairs wants to ease measures quickly to limit economic damage and reduce restrictions, while the Minister of Health is responsible for protecting the population, managing hospital capacity, and monitoring epidemiological trends. These competing interests create significant tension—this is just one example among many.”* (Cantonal representative)

Another barrier to communication, highlighted from different cantonal representatives, was the lack of clearly defined responsibilities. This ambiguity undermined the effectiveness of those involved and compromised the communication process within the institution.

*“At the beginning, there was also a bit of a conflict of competence within this communication structure. Is it now A, B or C? And who do I have to turn to and who says what now? And then there were also some external people who several responsibilities and it was not always clear, or they could not distinguish clearly between the responsibilities.”* (Cantonal representative)

The lack of resources in crisis communication is a critical barrier to effective communication within institutions. Without skilled professionals, navigating complex situations becomes challenging, leading to confusion and mistrust. However, with the right expertise, communication is clearer and more coordinated, improving crisis management. This barrier was also evident in discussions with diverse cantonal representatives, as in the following example.

*“But we were not practiced in truly joint communication, and we still are not. We have developed other resources. That is certainly a learning that we draw from this. At the cantonal level, we need a certain professionalization of crisis communication. We have now also hired a person to work on this.”* (Cantonal representative)

### Communication Across Public Health Institutions

In a federal state, internal communication also involves interactions across local, cantonal, and federal institutions. Thematic analysis revealed five facilitators—coordination, clear processes, active listening, mediation, and strong networks—and five barriers: role tensions, unclear responsibilities, lack of resources, and discrepancies among cantons and across governmental levels (Figure 1).

#### Facilitators to the Communication Across Public Health Institutions

Coordination was highlighted by many participants as a crucial facilitator because it enabled more effective communication across regional and cantonal institutions, as well as with federal authorities. This need for coordination becomes even more pronounced as the number of actors involved increases. Participants explained that when additional stakeholders—particularly municipalities—enter the communication chain, the demands for alignment grow substantially. Municipal staff are often the first point of contact for citizens, which means they must receive timely, consistent, and well-coordinated information to support their interactions with the public. This heightened need for alignment was described by one participant as follows:

*“but above all the help of municipalities is essential, but when more people are involved, the degree of alignment and coordination is also higher, because of the number of people who must get information and must stay up to date on that, because then they are the ones who have the citizen in front of them.”* (Cantonal representative)

Clear processes were also considered essential by our interviewees, as they helped prevent unpreparedness. By clarifying responsibilities, these processes supported coordinated actions and ensured a more organized response among public institutions.

*“We planned the first infection case meticulously. We also planned the first death in close coordination with the cantons. We considered every scenario: what if it happened at ten o’clock at night, or eight in the morning? What if it occurred in Ticino or in Bern, where access is different? What should we do—hold a press conference or issue a press statement? All of this was planned in advance. […]”* (Federal representative)

Because institutions operate within their own cultural contexts, participants noted that active listening plays a central role in enabling effective collaboration. This skill helps navigate the complexity of communication structures—where exchanges occur across multiple levels, committees, and institutional bodies. One participant illustrated how openness and accessibility underpin these interactions:

*“[…] The cantons are in a fairly active exchange, also with the federal government. But this happens at the most diverse levels. […] So there is basically a lot of exchange between the cantons, but on very different committees. And everyone has their own bodies where they exchange information. And basically, I consider the culture between the cantons to be open and accessible.”* (Cantonal representative)

Another critical communication competence that emerged in the Swiss federal context is mediation. Cantonal representatives emphasized that effective communication, especially with the Confederation, requires not only active listening but also a deep understanding of each institution’s perspective.

*“The Federal Council wants to close the ski terraces; the government wants to keep the ski terraces open. A very topical example, there are examples like this every day, but basically, yes. Our challenge is always to find a reasonable middle ground, so to speak, to create understanding.”* (Cantonal representative)

Many also deemed the social situation before the pandemic as important to support communication across public institutions. The presence of a good network and pre-existing direct relationships significantly facilitated communication, as these established connections allowed for more direct interactions and fostered trust. Moreover, a robust network also enhanced mediation efforts and enabled more effective joint actions.

*“We also have an exchange with all the cantonal doctors during the FOPH [i.e., Federal Office of Public Health] teleconference each week, then we have private exchanges with other cantonal doctors we know, we all know each other but we know some doctors better than others. […] We also try to make joint statements, all the Romande together […]”* (Cantonal representative)

#### Barriers to the Communication Across Public Health Institutions

As for communication within institutions, the tension between roles was also mentioned by representatives as a barrier to the communication among public health institutions.

*“And I believe that the independence of the scientific approach upheld within the FOPH and the Swiss state has important implications. One could simply look at a neighbouring country and follow the same decisions, but that is not how it works here. As evidence evolves, communication must adapt accordingly.”* (Federal representative)

According to many, another critical barrier to communication among the institutions was the lack of clearly defined responsibilities. Cantonal representatives reported instances where decisions were made and communicated without their input, leading to feelings of being overlooked and further complicating effective communication.

*“The GDK [i.e., The Swiss Conference of Cantonal Ministers of Public Health, the cantons' political coordination body for health policy] issued information about the canton of […] without any consultation with us. That was not good at all, because I had an excuse for another problem. And when the GDK communicated that, my excuse was almost blown. That was extremely bad. The information disclosure about things from our canton without consultation.”* (Cantonal representative)

The lack of resources in terms of cultural and linguistic competencies also emerged as a barrier to communication across public institutions. Due to Switzerland’s linguistic diversity, resources with specific linguistic skills were particularly important. The absence of such resources created a barrier, complicating effective communication across institutions.

*“And if you did not fully master the linguistic aspects, conflicts quickly emerged. People said: you are not paying attention to us, you have forgotten that we are also part of Graubünden—we would rather go to Ticino anyway. That was a significant challenge. […] At the same time, you still needed to obtain a consolidated view from other departments within the canton, which was practically impossible given the time pressure. I cannot ask my four colleagues in government for their opinion on every detail.”* (Cantonal representative)

Discrepancies among cantons were a significant barrier to communication across institutions. These differences often hindered productive discussions and exacerbated tensions with federal institutions. These discrepancies, potentially arising from cultural differences as well as contextual factors, were affecting the dialogue and impeding effective communication across institutions.

*“So it is taken into account to some extent. The problem is that the cantons are not strong enough to present a unified position to the Confederation. One canton agrees with the federal proposal, another thinks it goes too far, and a third believes it does not go far enough. This diversity of opinions ultimately strengthens the position of the Confederation, which can say: we listened, but since there is no consensus, we will proceed in this way. And that is a legitimate outcome.”* (Cantonal representative)

A final barrier was the discrepancies between different governmental levels (confederation and cantons), which impeded healthy communication across cantonal and federal institutional bodies. This sometimes resulted in fragmentation, disrupting cohesive communication across cantonal and federal institutional bodies.

*“Of course, we try to coordinate this as much as possible, so as not to create a federalist ping-pong where people cause each other problems or reproach each other. And that doesn't always work out. I think it works very well between HD and the GDK. But it doesn't always work between the federal government and individual cantons.”* (Cantonal representative)

## Discussion

This paper examined barriers and facilitators to communication within and across public institutions to inform future crisis strategies and complement existing models [4]. Federal representatives reported smooth internal communication, citing facilitators such as coordination and flexibility, and noted only minor cross-institutional barriers, mainly discrepancies among cantons. Cantonal representatives described a different picture: they experienced both barriers and facilitators within institutions, and, across institutions, pointed to significant barriers such as unclear responsibilities and inconsistencies across governmental levels, alongside facilitators like clear processes and strong networks. These contrasting views underscore the complexity of crisis communication. To prepare for future emergencies, institutions should systematically apply the identified facilitators to strengthen coordination and build on proven practices.

First, building communication capacity requires ongoing training to ensure that staff develop and maintain the competencies needed for effective interaction and crisis management. Participants repeatedly highlighted gaps in communication expertise—particularly in crisis communication—which reflects a broader preparedness challenge within public institutions. Despite this, communication processes within and across institutions have received limited attention in professional training studies [21, 22]. For example, reflections on past nuclear disasters highlight the difficulty of providing individualized risk communication and explaining radiation protection, stressing the need for training that covers evidence-based interventions, decision-making support, balancing risks and benefits, and addressing scientific uncertainty [21]. In today’s context, such training should be complemented with infodemic management [6, 23]. Facilitators identified in our study—including dedicated communication resources, skills such as active listening and mediation, and strong inter-institutional networks—further underscore the need for systematic and ongoing initiatives.

Furthermore, incorporating diverse perspectives through open dialogue with relevant stakeholders—such as public health authorities at different administrative levels, municipal representatives, healthcare professionals, and community organisations—is essential to ensure that all viewpoints are considered in decision-making [4]. Stakeholder engagement has proven valuable where scientific evidence or resources are limited, from local decisions [24] to global challenges such as the COVID-19 vaccine supply chain [25]. At the institutional level, networks, task forces, and interdisciplinary committees, together with active involvement of local stakeholders, can facilitate information sharing and coordination, yet remain underdeveloped and underused [26]. Our findings show that this lack of structured collaboration creates barriers across institutions, including role tensions, cantonal discrepancies, and misalignments between government levels. Such approaches are especially valuable in Switzerland’s complex federal and cultural context, where they can help ease tensions while respecting diversity.

Additionally, institutions must address barriers between cantonal and federal entities, as highlighted in this study. Clear communication protocols are crucial to streamline interactions and ensure consistency across governance levels. A centralized structure could enhance coordination and promote unified information dissemination. Yet integrating emergency risk communication guidelines depends on national contexts and may be shaped by leadership or organizational factors that can ease barriers such as legal constraints [3, 4]. In Switzerland, cantons hold substantial autonomy in public health, enabling locally tailored responses [16] but complicating centralized coordination in emergencies. Our findings show that, despite existing collaborations, discrepancies in mandates, practices, and resources across cantons hinder unified communication. Participants emphasized that these structural features—including varying crisis-communication capacities, differences in institutional culture, and the autonomy of cantonal decision-making—shape how communication unfolds in practice. For this reason, such structural realities must be taken into account when designing national crisis strategies. Participants suggested that national approaches should allow for flexible implementation at the cantonal level, strengthen mechanisms for coordination and information flow across federal, cantonal, and municipal actors, and ensure more equitable access to crisis-communication expertise and resources. These steps were seen as necessary to create national strategies that are both feasible and effective within Switzerland’s decentralised context.

Lastly, strengthening communication between cultural groups is vital. In Switzerland’s multicultural context, bridging differences within and across public health institutions promotes transparency, trust, and consistent communication for future emergencies. This mirrors patterns in public communication, where marginalized communities faced unequal access to COVID-19 information due to limited targeted messaging [27, 28]. Inclusive, culturally sensitive strategies—such as multilingual communication, adapted messaging, and collaboration with community representatives—are needed to ensure equitable information dissemination [29–31]. Such efforts foster the integration of disadvantaged groups and support transparent, unified communication [9]. They also align with facilitators identified in our study—coordination, mediation, active listening, and established networks—which, though present in some contexts, should be reinforced to build resilient and inclusive communication infrastructures.

This study has limitations inherent to its cross-sectional qualitative design, providing only a snapshot of crisis communication. However, data collection after two pandemic waves enabled more reflective accounts, offering insights to inform future preparedness. Pre-existing factors—such as the availability of dedicated crisis-communication staff, established coordination structures, and the robustness of internal communication channels—likely shaped each canton’s experience, and similar capacity-related constraints may also have influenced participants from federal departments. While some findings—such as the value of coordination mechanisms and communication capacity—may be relevant to other federal and multilingual systems, their transferability is limited by Switzerland’s specific governance structures, strong cantonal autonomy, and cultural–linguistic diversity, which may not be mirrored in more centralized or culturally homogeneous contexts.

The strength is that policy recommendations follow directly from the barriers and facilitators identified in the findings. Training addresses gaps in crisis-communication expertise, stakeholder engagement responds to limited collaboration, and clearer protocols and coordination mechanisms target inconsistencies between cantonal and federal practices. Cultural and linguistic challenges likewise point to the need for inclusive, multilingual strategies. Yet strong cantonal autonomy, differing capacities, and institutional cultures may hinder uniform implementation, making flexible, locally adaptable solutions essential in a decentralised system.

This study provides insights into communication dynamics within and across Swiss public health institutions during COVID-19. Drawing on federal and cantonal perspectives, it underscores the often-overlooked role of internal communication in emergency preparedness, whereas previous work has largely examined outward-facing communication with the public [20]. Effective communication—enabled by coordination, clear processes, and active listening—proved central to crisis management. The study identifies facilitators and barriers and offers recommendations, including clear protocols, centralized structures, continuous training, and attention to cultural and linguistic diversity. While reflecting a specific moment, these findings offer enduring lessons for strengthening crisis communication and institutional preparedness.

## Data Availability

The dataset used and analyzed during the current study are available from the corresponding author on reasonable request.

## References

[1] Cui L . Risk Communication in the Post-Fukushima Era. Radiat Med Prot (2021) 2(2):79–82. 10.1016/j.radmp.2021.04.004

[2] Seiler M Staubli G Hoeffe J Gualco G Manzano S Goldman RD . A Tale of Two Parts of Switzerland: Regional Differences in the Impact of the COVID-19 Pandemic on Parents. BMC Public Health (2021) 21(1):1275. 10.1186/s12889-021-11315-5 34193102 PMC8242280

[3] Schnabel J Freiburghaus R Hegele Y . Crisis Management in Federal States: The Role of Peak Intergovernmental Councils in Germany and Switzerland During the COVID-19 Pandemic. dms – der moderne staat – Z für Public Pol Recht Management (2022) 15(1):42–61. 10.3224/dms.v15i1.10

[4] Jha A Lin L Short SM Argentini G Gamhewage G Savoia E . Integrating Emergency Risk Communication (ERC) Into the Public Health System Response: Systematic Review of Literature to Aid Formulation of the 2017 WHO Guideline for ERC Policy and Practice. PLOS ONE (2018) 13(10):e0205555. 10.1371/journal.pone.0205555 30379900 PMC6209198

[5] Reynolds BW Seeger M . Crisis and Emergency Risk Communication as an Integrative Model. J Health Commun (2005) 10(1):43–55. 10.1080/10810730590904571 15764443

[6] Rubinelli S Purnat TD Wilhelm E Traicoff D Namageyo-Funa A Thomson A WHO Competency Framework for Health Authorities and Institutions to Manage Infodemics: Its Development and Features. Hum Resour Health (2022) 20(1):35. 10.1186/s12960-022-00733-0 35525924 PMC9077350

[7] Borges do Nascimento IJ Pizarro AB Almeida JM Azzopardi-Muscat N Gonçalves MA Björklund M Infodemics and Health Misinformation: A Systematic Review of Reviews. Bull World Health Organ (2022) 100(9):544–61. 10.2471/BLT.21.287654 36062247 PMC9421549

[8] Fiordelli M Rubinelli S Diviani N . Acceptance of Public Health Measures During the COVID-19 Pandemic: A Cross-Sectional Study of the Swiss Population’s Beliefs, Attitudes, Trust, and Information-Seeking Behavior. Int J Public Health (2023) 68:1605982. 10.3389/ijph.2023.1605982 37408794 PMC10318558

[9] Zimmermann BM Fiske A McLennan S Sierawska A Hangel N Buyx A . Motivations and Limits for COVID-19 Policy Compliance in Germany and Switzerland. Int J Health Policy Manag (2021) 11(8):1342–53. 10.34172/ijhpm.2021.30 33949815 PMC9808338

[10] Selby K Durand MA Gouveia A Bosisio F Barazzetti G Hostettler M Citizen Responses to Government Restrictions in Switzerland During the COVID-19 Pandemic: Cross-Sectional Survey. JMIR Formative Res (2020) 4(12):e20871. 10.2196/20871 33156809 PMC7717891

[11] Schnabel J Hegele Y . Explaining Intergovernmental Coordination During the COVID-19 Pandemic: Responses in Australia, Canada, Germany, and Switzerland. Publius: The J Federalism (2021) 51(4):537–69. 10.1093/publius/pjab011

[12] Willi Y Nischik G Braunschweiger D Pütz M . Responding to the COVID-19 Crisis: Transformative Governance in Switzerland. Tijdschrift voor Economische en Sociale Geografie (2020) 111(3):302–17. 10.1111/tesg.12439 32836490 PMC7323205

[13] Dworakowski O Meier T Mehl MR Pennebaker JW Boyd RL Horn AB . Comparing the Language Style of Heads of State in the US, UK, Germany and Switzerland During COVID-19. Sci Rep (2024) 14(1):1708. 10.1038/s41598-024-51362-7 38242954 PMC10799077

[14] De Pietro C Camenzind P Sturny I Crivelli L Edwards-Garavoglia S Spranger A Switzerland: Health System Review. Health Syst Transit (2015) 17(4):1–288, xix. Available online at: https://iris.who.int/server/api/core/bitstreams/a3a599b2-18d2-43ac-ad07-2fb04820e486/content. 26766626

[15] OECD. The Performance of the Swiss Health System: Effectiveness and Quality. Paris: OECD (2006). Available online at: https://www.oecd-ilibrary.org/social-issues-migration-health/oecd-reviews-of-health-systems-switzerland-2006/the-performance-of-the-swiss-health-system_9789264025837-4-en (Accessed August 16, 2024).

[16] Jedwab J Kincaid J . 5 Political Trust in Switzerland: Again a Special Case? In: Identities, Trust, and Cohesion in Federal Systems. Montreal: McGill-Queen’s University Press (2019). p. 115–45. Available online at: https://www.degruyterbrill.com/document/doi/10.1515/9781553395379-006/pdf?licenseType=restricted (Accessed April 14, 2025).

[17] Bangerter A Krings F Mouton A Gilles I Green EGT Clémence A . Longitudinal Investigation of Public Trust in Institutions Relative to the 2009 H1N1 Pandemic in Switzerland. PLoS ONE 7 e49806. 10.1371/journal.pone.0049806 PMC3504102 23185444

[18] Smith JA Larkin M Flowers P . Interpretative Phenomenological Analysis: Theory, Method and Research. (2021). p. 1–100.

[19] Clarke V Braun V . Thematic Analysis. The J Positive Psychol (2017) 12(3):297–8. 10.1080/17439760.2016.1262613

[20] Rubinelli S Häfliger C Fiordelli M Ort A Diviani N . Institutional Crisis Communication During the COVID-19 Pandemic in Switzerland. A Qualitative Study of the Experiences of Representatives of Public Health Organizations. Patient Education Couns (2023) 114:107813. 10.1016/j.pec.2023.107813 37247524 PMC10207862

[21] Ohtsuru A Tanigawa K Kumagai A Niwa O Takamura N Midorikawa S Nuclear Disasters and Health: Lessons Learned, Challenges, and Proposals. Lancet (2015) 386(9992):489–97. 10.1016/S0140-6736(15)60994-1 26251394

[22] Murakami M Kumagai A Ohtsuru A . Building Risk Communication Capabilities Among Professionals: Seven Essential Characteristics of Risk Communication. Radiat Prot Dosimetry (2018) 182(1):120–7. 10.1093/rpd/ncy140 30165706

[23] Purnat T Bertrand-Ferrandis C Yau B Ishizumi A White B Briand S Training Health Professionals in Infodemic Management to Mitigate the Harm Caused by Infodemics. Eur J Public Health (2022) 32(Suppl. ment_3):ckac129–324. 10.1093/eurpub/ckac129.324

[24] Hoover AG Heiger-Bernays W Ojha S Pennell KG . Balancing Incomplete COVID-19 Evidence and Local Priorities: Risk Communication and Stakeholder Engagement Strategies for School Re-Opening. Rev Environ Health (2021) 36(1):27–37. 10.1515/reveh-2020-0092 33001857 PMC7933073

[25] Kazancoglu Y Sezer MD Ozbiltekin-Pala M Kucukvar M . Investigating the Role of Stakeholder Engagement for More Resilient Vaccine Supply Chains During COVID-19. Oper Manag Res (2022) 15(1):428–39. 10.1007/s12063-021-00223-x

[26] Aguilera B Donya Rs. Vélez CM Kapiriri L Abelson J Nouvet E Stakeholder Participation in the COVID-19 Pandemic Preparedness and Response Plans: A Synthesis of Findings from 70 Countries. Health Policy (2024) 142:105013. 10.1016/j.healthpol.2024.105013 38401332

[27] Austin EW Austin BW Willoughby JF Amram O Domgaard S . How Media Literacy and Science Media Literacy Predicted the Adoption of Protective Behaviors Amidst the COVID-19 Pandemic. J Health Commun (2021) 26(4):239–52. 10.1080/10810730.2021.1899345 33928871

[28] Viswanath K Lee EWJ Pinnamaneni R . We Need the Lens of Equity in COVID-19 Communication. Health Commun (2020) 35(14):1743–6. 10.1080/10410236.2020.1837445 33106029

[29] Venkatraman K Manoharan A . Public Engagement as the Fifth Dimension of Outbreak Communication: Public’s Perceptions of Public Health Communication During COVID-19 in India. Health Commun (2023) 38(2):285–97. 10.1080/10410236.2021.1950294 34294016

[30] Kalocsányiová E Essex R Poulter D . Risk and Health Communication During Covid-19: A Linguistic Landscape Analysis. Health Commun (2023) 38(6):1080–9. 10.1080/10410236.2021.1991639 34696637

[31] Kalocsányiová E Essex R Fortune V . Inequalities in Covid-19 Messaging: A Systematic Scoping Review. Health Commun (2023) 38(12):2549–58. 10.1080/10410236.2022.2088022 35850593

