# Supplementary material for: Effective Communication Within and Across Public Health Institutions: A Qualitative Study in Switzerland
Source: Int J Public Health. 2026 Jan 8;70:1609055. doi: 10.3389/ijph.2025.1609055 (PMC12823545; doi:10.3389/ijph.2025.1609055)
Supplement: Supplementary file 1 [file Supplementaryfile1.docx]

**Guide for Semi-Structured Interviews**

## Warming up

| 5 Mins  Personal ***professional*** experience of institutional communication with the pandemic |
| --- |

- ***QUESTIONS:***

1. *Do you remember when you first realized that COVID was a problem?*
2. *At what point did you — in your professional role — or your institution first communicate / engage with the public about the pandemic?*
3. *What was your personal professional experience with your institutions’ communicational efforts during the different phases of the pandemic, starting with the first action of you / your institution you just talked about? What was easiest / most difficult aspect (things to inform people)? (a: at the beginning of the pandemic; b: after a month; c: during “lockdown”; d: when it seemed that the first wave was ending; e: after the first wave; f: beginning / during the second wave; g: currently*
4. *In your perception, what is different for the first and second wave?*

- ***ASPECTS OF INTEREST:***

Specific reactions /changes in strategies throughout the pandemic and due to central events

- - First wave:
    - First knowledge about pandemic
    - (First and ongoing) measures
    - Begin of lockdown
    - During lockdown
    - Post lockdown (reopening/now; ensuring motivation and compliance)
  - Second wave: see above

| 5 Mins  Overall strategy of communication |
| --- |

- ***QUESTIONS:***

1. *How did you plan communication during this time?*
2. *Did it change at some point?*
3. *What, concretely, was communicated and how?*
4. *What guided / influenced / inspired different communicative actions?*

- ***ASPECTS OF INTEREST:***

What tools are used?

- - Guidelines
  - Frameworks
  - Models
  - Best practices
  - (Common knowledge / past experiences?

| 3 Mins  Planning & Research |
| --- |

***QUESTION:***

1. *How do you get the necessary information about the pandemic? Are there any strategies or tools you use for information screening?*
2. *What where your most important sources for information?*

- ***ASPECTS OF INTEREST:***
  - Screening information
  - Sources of information
  - Identification of dis/misinformation

| 5 Mins  Development (Strategy & Messages / Material) |
| --- |

***QUESTION:***

1. *How did you plan your communicative actions?*
2. *How did you identify information needs in your target population?*
3. *What exactly was communicated? How were messages designed? What did guide those decisions?*
4. *What did you want to achieve with your communicational efforts?*
5. *Do you conduct pre-evaluation or pretesting of the messages or information?*

- ***ASPECTS OF INTEREST:***
  - Strategic decisions / approach to communication
  - If and how decide what to communicate / content
  - How to communicate (tailoring)
  - Which determinants / factors /… get targeted?
    - Providing information
    - Growing knowledge
    - Influencing beliefs / attitudes
    - Risk perception (correct)
    - Empowering / improving efficacy (self / response) perceptions
    - Building / maintaining / restore trust
  - Actions to pretest messages for effects and acceptance among target populations?

| 5 Mins  Implementation |
| --- |

- ***QUESTION:***

1. *What are your target groups? How were they chosen and why?*
2. *Which channels were chosen to disseminate the information and why? (Did you choose channels as a function of the targeted audience?)*
3. *How did your / your communicative efforts (e.g., with regard to channels / target groups) develop over time?*
4. *Do you have a specific budget allocated to this kind of communication?*

- ***ASPECTS OF INTEREST:***
  - Choice of channels (multi-channel strategy)
  - Expansion of communicative efforts over time
  - Target groups
  - Costs / budgetarily issues

| 5 Mins  Monitoring & Evaluation |
| --- |

- ***QUESTION:***

1. *Are the communicative actions evaluated afterwards?*
2. *If so: Do you have a system in place for evaluating the impact of your institution health messages?
   E.g, specifically: In order to understand determinants of individual and community behavior, do you measure attitudes, knowledge, beliefs, health literacy and social norms about COVID-19 and in light of the information that you deliver as a health institution?*
3. *Is feedback from your target population considered during or after the dissemination? How?*

- ***ASPECTS OF INTEREST:***
  - Is there any evaluation of outcomes?
  - What is the approach to evaluation?
    - Before implementation / pretesting?
    - Ongoing?
    - Afterwards?
  - Feedback
    - Feedback channels
    - Taking into account of feedback (also for future activities)
    - Learning process
    - Participatory / community feedback mechanisms for community-led responses

| Max. 10 Mins  Major learnings |
| --- |

- ***QUESTION:***

1. *Looking back now (maybe also explicitly talked about before already)… what are, in your personal professional opinion, the differences between the first and the second wave in terms of communication?*
   1. *What was easier or more difficult*
   2. *Different priorities / topics*
   3. *Reaction times*
   4. *…*
2. *What competences did you feel were valuable to communicate during the pandemic?*
3. *Did you feel empowered after the first wave? How about now? (Did it change between the first and the second wave?)*
4. *What resources would you think are needed to enable effective communication?*
   1. *Training*
   2. *Financial*
   3. *Human*
   4. *Knowledge*
5. *What would you say are the major aspects of your work the pandemic has influenced and that will also influence your work in the future? (Learnings)*
6. *Are there things your organization has achieved that you think are important?*

- ***ASPECTS OF INTEREST:***
  - (Major) achievements
  - (Major) problems

## Macro / external perspective 🡪 transition

| 10 Mins  From institutional level to a broader context |
| --- |

***QUESTION:***

1. *From the perspective of your institution, what role did other disseminators of information / major communicative actors play for your institution or your work? (perceptions about problems / advantages / achievements with respect to external communications that did not come from the institution itself)*
2. *How did the situations in*
   1. *other cantons*
   2. *Switzerland as a country*
   3. *other countries*

*affect your work?*

*The WHO has identified* ***infodemic*** *as one major problem of this health crisis. Did you experience any problems with this during the crisis?*

- 1. *dis- / misinformation?*
  2. *fake news?*
  3. *conspiracy theories?*

*(An* ***infodemic*** *is an overabundance of information, both online and offline. It includes deliberate attempts to disseminate wrong information to undermine the public health response and advance alternative agendas of groups or individuals. Mis- and disinformation can be harmful to people’s physical and mental health; increase stigmatization; threaten precious health gains; and lead to poor observance of public health measures, thus reducing their effectiveness and endangering countries’ ability to stop the pandemic)*

1. *What was your strategy to deal with the* ***infodemic****, e.g., identify and deal with dis-/misinformation?*
2. *Did your institution make changes to better deal with these issues? How?*

- ***ASPECTS OF INTEREST:***
  - Perceived challenges with regard to
    - Other cantons / problems due to federalism (differences of measures, communication, … in other cantons)
    - On a national level (BAG, government)
    - Other countries / international level
  - Issues with infodemic / dis- or misinformation / fake-news / conspiracy theory
    - Identification
    - Strategy
    - Measures taken
    - (Perceived) outcomes / success
    - Competences / tools usefeul in order to deal with infodemic

| Open  Wrap up |
| --- |

Is there any other topic/issue that you would like to address about institutional communication, engagement, and management of the COVID-19 pandemic?
